# Preoperative HALP Score as a Marker of Tumor Aggressiveness and Survival in Surgically Treated Soft Tissue Sarcoma: A Retrospective Cohort Study

**DOI:** 10.3390/jcm15083044

**Published:** 2026-04-16

**Authors:** Hüseyin Pülat, Oğuzhan Söyler, Ünal Öner, Deniz Öztaşan, Cüneyt Akyüz, Cemil Yüksel

**Affiliations:** Department of Surgical Oncology, Mersin City Training and Research Hospital, University of Health Sciences, Mersin 33230, Türkiye; drhpulat@gmail.com (H.P.); droguzhansoyler@gmail.com (O.S.); unaloner90@gmail.com (Ü.Ö.); denizoztasan@gmail.com (D.Ö.); cuneyt_akyuz@yahoo.com (C.A.)

**Keywords:** soft tissue sarcoma, HALP score, systemic inflammation, overall survival

## Abstract

**Objectives:** Soft tissue sarcomas (STS) are biologically heterogeneous malignancies with unpredictable clinical behavior. Although tumor size, histological grade, and surgical margin status remain the main determinants of prognosis, additional biomarkers that integrate tumor biology and host-related factors are needed. The hemoglobin × albumin × lymphocyte/platelet (HALP) score reflects systemic inflammation and nutritional status. This study aimed to evaluate the association between preoperative HALP score and oncological as well as surgical outcomes in patients undergoing curative resection for STS. **Materials and Methods:** A retrospective cohort study was conducted including 46 consecutive patients who underwent surgery for STS between 2017 and 2025. HALP scores were calculated using preoperative laboratory parameters, and patients were stratified into low- and high-HALP groups according to the cohort median (24.9). Overall survival (OAS) and disease-free survival (DFS) were analyzed using the Kaplan–Meier method and Cox proportional hazards models. Surgical margin status and postoperative complications were also compared. **Results:** Patients with low HALP scores had significantly larger tumors, higher rates of non-R0 resection, and increased major complications (*p* < 0.05). Recurrence and mortality were more frequent in the low-HALP group. Kaplan–Meier analysis demonstrated significantly shorter OAS (log-rank *p* = 0.0034) and DFS (log-rank *p* = 0.0318) in patients with low HALP scores. In univariate Cox analysis, HALP was significantly associated with survival outcomes; however, in multivariate analysis, histological grade and surgical margin status remained independent prognostic factors, while HALP lost independent significance. **Conclusions:** A low preoperative HALP score is associated with aggressive tumor characteristics, increased surgical morbidity, and poorer survival in STS patients. Although HALP did not retain independent significance in multivariable analysis, its strong association with tumor aggressiveness and survival suggests that it may reflect the systemic manifestation of high-risk tumor biology. As a simple and cost-effective biomarker derived from routine laboratory parameters, HALP may support preoperative risk stratification and help identify patients with biologically aggressive disease.

## 1. Background

Soft tissue sarcomas (STS) are rare and biologically heterogeneous malignancies originating from mesenchymal tissue and include more than 50 histological subtypes [1]. In STS, the cornerstone of curative treatment is surgical resection; however, local recurrence and distant metastasis rates remain high. Therefore, identifying clinical and biological markers that can predict the prognosis in STS is of great importance.

Many prognostic factors associated with overall survival have been identified. Tumor size, histological grade, localization and surgical margin status are among these [2,3]. While these factors are valuable in clinical decision-making, there is still a need for additional biomarkers that can make patient-specific risk stratification more precise.

In recent years, it has been shown that tumor biology is closely related not only to tumor-specific characteristics but also to systemic inflammation and nutritional status [4]. Studies have found that blood cells such as platelets, monocytes, neutrophils and lymphocytes are associated with and important for tumor proliferation, invasion and distant organ metastasis [5]. By comparing different inflammatory markers, information has been obtained about the course of cancer. Various inflammatory markers such as neutrophil–lymphocyte ratio, platelet–lymphocyte ratio and systemic inflammation response index have been used for prognostic purposes in different cancer types [6,7].

The HALP score (HALP score = hemoglobin × lymphocyte × albumin/platelet), an immuno-nutritional biomarker developed by Chen et al. in 2015 to predict prognosis in gastric cancer, has been used in subsequent years to predict a range of clinical outcomes in various benign and malignant clinical conditions [8]. In subsequent years, the HALP score was evaluated as a prognostic marker in various solid tumors.

The first study on the HALP score in the context of retroperitoneal soft tissue sarcoma was published by Matsui et al. in 2022, but the HALP score did not show any prognostic value for overall survival [1]. However, data on the prognostic role of preoperative HALP score in extremity and trunk-located STS patients are limited. In this study, we aimed to evaluate the effect of preoperative HALP score as a prognostic factor on overall survival in patients who underwent surgery due to STS.

## 2. Methods

This retrospective single-center observational cohort study included consecutive patients who underwent curative resection at our clinic between January 2017 and April 2025 and were diagnosed histopathologically with soft tissue sarcoma.

Inclusion criteria are as follows: patient must be over 18 years of age, have histopathologically confirmed primary soft tissue sarcoma, have undergone curative surgical resection, and have all preoperative laboratory and radiological data available, as well as complete follow-up data.

Exclusion criteria include patients who have previously undergone cancer surgery, have metastatic disease, have received chemotherapy or radiotherapy for another reason, have chronic liver or kidney failure, have a history of hematological or chronic inflammatory diseases, and have incomplete laboratory or follow-up data.

Demographic, clinicopathological, and perioperative data were obtained from the hospital information management system and electronic patient files.

The variables collected were: age and gender, tumor size, grade, resection status, postoperative complications (Clavien–Dindo classification), recurrence, mortality, disease-free survival, and overall survival. Histological subtypes were recorded based on pathological reports. All tumors included in the study were intra-abdominal soft tissue sarcomas. Due to the heterogeneous distribution of histological subtypes and the limited number of patients within each subgroup, no subtype-specific statistical analysis was performed. (Table 1).

Major complications were defined as Clavien–Dindo ≥ 3.

The hemoglobin–albumin–lymphocyte–platelet (HALP) score was calculated using the following formula. The HALP score was calculated based on preoperative hemoglobin (g/dL), albumin (g/dL), lymphocyte count (103/µL), and platelet count (103/µL) values as follows: HALP = 100 × [Hb × Alb × lymphocyte/PLT]. Laboratory values were standardized to ensure unit consistency. Based on the median HALP value of the study cohort (24.9), patients were divided into two groups: low HALP (<24.9) and high HALP (≥24.9).

The primary endpoints of the study were overall survival (OAS) and disease-free survival (DFS), while the secondary endpoints were postoperative complications and major complications. OAS is defined as the time elapsed from the date of surgery to death from any cause. DFS was defined as the time from the date of surgery to recurrence or death, whichever occurred first.

### Statistical Analysis

Statistical analyses were performed using IBM SPSS Statistics for Windows, Version 25.0 (IBM Corp., Armonk, NY, USA). The distribution of continuous variables was assessed using the Shapiro–Wilk test. Variables not normally distributed were presented as median (interquartile range, IQR), and between-group comparisons were performed using the Mann–Whitney U test. Categorical variables were expressed as number and percentage (%) and compared using the chi-square test or Fisher’s exact test, as appropriate. Overall survival (OAS) and disease-free survival (DFS) were analyzed using the Kaplan–Meier method, and groups were compared with the log-rank test. Cox proportional hazards regression analysis was used to evaluate prognostic factors. The proportional hazards assumption was tested using Schoenfeld residuals, confirming that model assumptions were met. Due to the limited number of events, to avoid overfitting, only a limited number of variables that were clinically relevant and had *p* < 0.10 in univariate analysis were included in the multivariate model. Results were reported as hazard ratios (HRs) with 95% confidence intervals (CIs). Statistical significance was set at *p* < 0.05.

The study was approved by the Institutional Ethics Committee of SBÜ Mersin City Training and Research Hospital (Approval No: 39/2025) and conducted in accordance with the Declaration of Helsinki.

## 3. Results

A total of 46 patients were included in the study. The baseline clinicopathological characteristics of the study cohort are presented in Table 2. The median age was 58 years (IQR: 45–67), and 58.7% of patients were male. The median tumor size was 115 mm (IQR: 85–180). Grade 3 tumors were present in 45.6% of patients. The R0 resection rate was 52.2%. The median follow-up was 20 months (IQR: 14–51). The reverse Kaplan–Meier method was used to estimate median follow-up. During follow-up, recurrence occurred in 23 patients (50%), and death was observed in 23 patients (50%). The median HALP score of the cohort was 24.9 (IQR: 18.6–34.2). Based on the median HALP value, patients were divided into two groups (n = 23 low HALP, n = 23 high HALP). Clinicopathological comparisons according to HALP groups are presented in Table 3. Tumor size was significantly larger in the low-HALP group: low HALP, median 140 mm (IQR: 112–210) vs. high HALP, median 90 mm (IQR: 75–130) (*p* = 0.0107).

The proportion of patients without R0 resection was significantly higher in the low-HALP group: low HALP, 69.6% vs high HALP, 26.1% (*p* = 0.0119). Major complications (Clavien–Dindo ≥ 3) were observed only in the low-HALP group: low HALP, 34.8% vs. high HALP, 0% (*p* = 0.0038). Recurrence (65.2% vs. 34.8%, *p* = 0.048) and mortality (69.6% vs. 30.4%, *p* = 0.017) rates were also higher in the low-HALP group.

### 3.1. OAS

**Figure 1:**
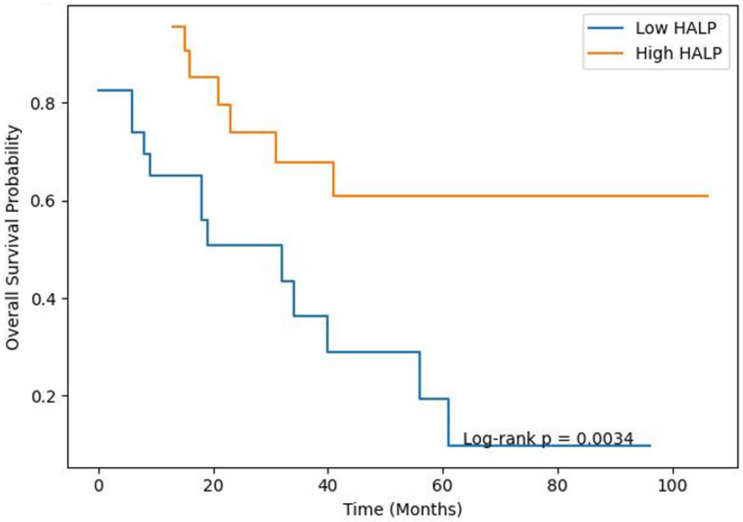
Kaplan–Meier curves for overall survival according to HALP group.

In the Kaplan–Meier analysis, overall survival was significantly shorter in the low-HALP group (log-rank *p* = 0.0034) (Figure 1). Median OAS was 32 months in the low-HALP group, whereas it was not reached in the high-HALP group.

In univariate Cox analysis, a high HALP score was associated with a significantly reduced risk of mortality (HR 0.285, 95% CI 0.116–0.700; *p* = 0.006). In addition, higher tumor grade (HR 2.73, 95% CI 1.50–4.98; *p* = 0.001) and non-R0 resection (HR 4.04, 95% CI 1.58–10.32; *p* = 0.003) were significantly associated with mortality. In multivariate Cox analysis, high grade remained an independent prognostic factor (HR 2.23, 95% CI 1.18–4.21; *p* = 0.014), whereas the HALP score lost its independent significance when grade and resection status were included in the model (HR 0.82, 95% CI 0.23–2.87; *p* = 0.785). Non-R0 resection showed borderline significance (HR 2.76, 95% CI 0.89–8.56; *p* = 0.083). The results of the univariate and multivariate Cox analyses are presented in Table 4.

### 3.2. DFS

**Figure 2:**
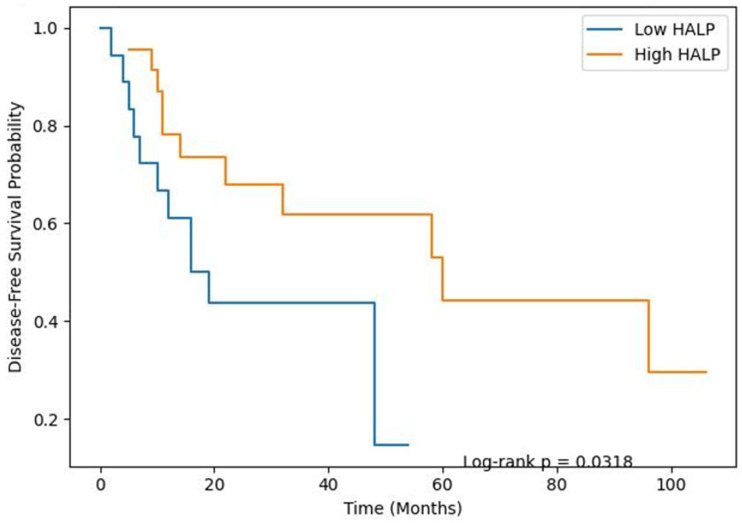
Kaplan–Meier curves for disease-free survival according to HALP group.

Disease-free survival was significantly shorter in the low-HALP group (log-rank *p* = 0.0318) (Figure 2). Median DFS was 16 months in the low-HALP group and 60 months in the high-HALP group.

In univariate Cox analysis, a high HALP score was associated with a reduced risk of recurrence (HR 0.389, 95% CI 0.158–0.960; *p* = 0.040). Higher tumor grade and non-R0 resection were also significantly associated with DFS. In multivariate analysis, the HALP score lost its independent prognostic value (HR 0.74, 95% CI 0.28–1.95; *p* = 0.559). In contrast, high grade (HR 1.94, 95% CI 1.05–3.59; *p* = 0.033) and non-R0 resection (HR 7.22, 95% CI 2.19–23.77; *p* = 0.002) remained independent prognostic factors.

Kaplan–Meier analysis demonstrating overall survival (OAS) stratified by preoperative HALP score. Patients were categorized into low-HALP (<24.9) and high-HALP (≥24.9) groups based on the cohort median. The low-HALP group exhibited significantly poorer overall survival compared to the high-HALP group (log-rank *p* = 0.0034). Shaded areas represent 95% confidence intervals. The number of patients at risk at selected time points is displayed below the plot.

Kaplan–Meier analysis of disease-free survival (DFS) according to preoperative HALP score. Patients were stratified using the median HALP value (24.9). The low-HALP group demonstrated significantly shorter DFS compared to the high-HALP group (log-rank *p* = 0.0318). Shaded areas indicate 95% confidence intervals. The number of patients at risk is shown beneath the graph.

## 4. Discussion

Soft tissue sarcomas are a type of cancer with a difficult prognosis to predict due to their biodiversity and varied clinical course. Current guidelines state that the key determinants of prognosis are tumor size, histological grade, and resectability [9]. In this context, investigating preoperative biomarkers that may affect prognosis is of clinical importance.

The limited evidence regarding the prognostic role of HALP in soft tissue sarcomas prompted us to investigate its clinical relevance in a surgically treated STS cohort [1]. This study demonstrated that preoperative HALP score was significantly associated with both overall survival (OAS) and disease-free survival (DFS), but this association lost its independence in multivariate analysis. In patients with a low HALP score, larger tumor size, a higher rate of non-R0 resection, increased major complications, and higher recurrence and mortality rates were observed. In Kaplan–Meier analysis, both OAS and DFS were found to be significantly shorter.

The HALP score is a combined index of hemoglobin, albumin, lymphocyte, and platelet values that reflects systemic inflammation, nutritional status, and immune response. In recent years, it has been shown that a low HALP score is associated with poor survival in various solid tumors [10,11]. Anemia, hypoalbuminemia, and lymphopenia are considered indicators of tumor burden, catabolic state, and systemic inflammatory response. In particular, anemia has been reported to be associated with poor prognosis in STS at the level of systematic reviews [12]. Within this biological framework, a low HALP score likely reflects both aggressive tumor biology and reduced physiological reserve. Furthermore, closer preoperative assessment of patients with a low HALP score may be recommended. Approaches such as nutritional support and correction of anemia in these patients will indirectly improve long-term outcomes by reducing perioperative risks [13].

This association is also biologically plausible. Systemic inflammation is known to play a central role in cancer progression by modulating the tumor microenvironment, promoting tumor cell proliferation, invasion, angiogenesis and immune evasion [14]. In addition, inflammatory mediators and immune-related processes contribute to tumor growth and metastatic potential, particularly through interactions between tumor cells and host immune components. In soft tissue sarcomas, the tumor microenvironment is highly complex and characterized by heterogeneous immune cell infiltration, cytokine signaling and immune checkpoint activity, all of which may significantly influence tumor behavior and clinical outcomes [15]. Moreover, recent genomic and translational studies suggest that immune system activity and tumor–host interactions are closely linked to treatment response and disease aggressiveness in sarcomas [16]. Furthermore, nutritional status represents another critical determinant of cancer prognosis. Markers reflecting nutritional and inflammatory balance such as albumin-related indices are associated with systemic resilience and the ability to tolerate oncologic stress and major surgical interventions [14]. Therefore, a low HALP score may reflect a combined state of systemic inflammation, immune dysregulation and impaired nutritional status, all of which are biologically linked to more aggressive behavior and poorer oncological outcomes.

The prognostic role of systemic inflammation indices in soft tissue sarcomas has been investigated in recent years. It has been reported that indices such as SII and PNI may have prognostic value in STS [17,18]. However, the independence of these indices is inconsistent, and in most studies, tumor size and grade remain the dominant prognostic factors. Our study contributes to the literature by demonstrating that HALP is associated with clinicopathological high-risk features in STS. In the present study, the HALP score was significantly associated with both overall survival and disease-free survival in Kaplan–Meier and univariate analyses. Although HALP did not retain independent significance after adjustment for tumor grade and surgical margin status, its strong association with adverse clinicopathological characteristics suggests that HALP may reflect the systemic manifestation of aggressive tumor biology rather than acting as an isolated prognostic determinant. These findings indicate that HALP may capture the interaction between tumor aggressiveness and host-related biological vulnerability, including systemic inflammation, nutritional status, and immune competence. This finding is consistent with the literature showing that the main factors determining prognosis in STS are still tumor biology and surgical margin status [19].

In sarcomas, the cornerstone of curative treatment is R0 resection. Resectability and local control are critical for long-term survival. Therefore, in the preoperative period, predicting patient and tumor characteristics that may affect surgical margins is clinically valuable. In our study, the finding that a low HALP score was associated with a higher rate of non-R0 resection suggests an indirect relationship between the HALP score and surgical outcomes.

The association between a low HALP score and an increased rate of major complications is clinically significant. This suggests that a low HALP score may reflect not only oncological outcomes but also perioperative physiological reserve. Given that STS surgery typically involves wide resection, the effects of nutritional status and anemia on postoperative morbidity are well recognized. Therefore, HALP can be a practical tool in the preoperative period for: identifying at-risk patients, nutritional optimization, anemia management, and prehabilitation programs [1]. However, these suggestions need to be validated through prospective studies.

The HALP score is an inexpensive, readily available, and easily calculated index derived from routine laboratory parameters. Our findings demonstrate that a low HALP score is consistently associated with larger tumors, higher rates of non-R0 resection, increased postoperative complications, and poorer survival outcomes. These observations support the concept that HALP may serve as a practical biomarker reflecting both tumor aggressiveness and the host’s systemic inflammatory and nutritional status. Consequently, preoperative HALP assessment may help identify patients with a biologically aggressive disease and increased surgical risk.

There is no universally accepted cutoff value for HALP in the literature. Although the reported cutoff values vary widely across studies, values in the range of 20–35 have most commonly been used [20,21]. In our study, the median value was calculated as 24.9, which is consistent with the range defined as low HALP in the literature. This heterogeneity highlights the need to establish standardized HALP cutoff values across different populations.

This study has some limitations. The retrospective and single-center design, limited sample size (n = 46), short median follow up duration and histological subtype heterogeneity restrict the generalizability of the results. In addition, due to the heterogeneous distribution of histological subtypes and the limited number of patients within each subgroup, a subtype-specific analysis of the association between HALP score and histological subtype could not be reliably performed. Furthermore, cytogenetic and molecular data were not consistently available in this retrospective cohort, particularly in earlier cases where comprehensive molecular profiling was not routinely performed and molecular and immunohistochemical evaluations were reported by different pathologists, which may introduce variability in interpretation and some findings were not systematically included in pathology reports. Therefore, a reliable analysis of the association between HALP score and molecular features could not be conducted. Furthermore, HALP is a dynamic parameter and can be affected by fasting status, blood sampling time, and nutritional variables. Future prospective and multicenter studies will provide a clearer picture of the prognostic role of HALP in STS and incorporating standardized molecular profiling may further clarify whether HALP score correlates with specific genetic or biological tumor subtypes. It is also important to analyze HALP as a continuous variable and to test different cutoff values.

## 5. Conclusions

In patients undergoing surgery for soft tissue sarcoma, a low preoperative HALP score is associated with more aggressive tumor characteristics, increased postoperative complications, and significantly worse overall and disease-free survival. Although HALP did not remain an independent prognostic factor after adjustment for established predictors such as tumor grade and surgical margin status, it demonstrated a strong association with adverse oncological and surgical outcomes. These findings suggest that HALP may reflect the systemic expression of aggressive tumor biology and host vulnerability. As a simple and cost-effective biomarker derived from routine laboratory tests, the HALP score may contribute to improved preoperative risk stratification in patients with soft tissue sarcoma.

## Figures and Tables

**Table 1:** Distribution of histological subtypes and tumor location (n = 46).

Histological Subtype	n (%)
Liposarcoma	19 (41.3)
Leiomyosarcoma	10 (21.7)
Undifferentiated pleomorphic sarcoma	9 (19.6)
Fibrosarcoma	4 (8.7)
Rhabdomyosarcoma	2 (4.3)
Extraskeletal Ewing sarcoma	1 (2.2)
Angiosarcoma	1 (2.2)

**Table 2:** Baseline characteristics of the study population (n = 46).

Variable	Overall (n = 46)
Age, median (IQR)	58 (45–67)
Male sex, n (%)	27 (58.7)
Tumor size (mm), median (IQR)	115 (85–180)
Grade 1, n (%)	8 (17.4)
Grade 2, n (%)	17 (37.0)
Grade 3, n (%)	21 (45.6)
R0 resection, n (%)	24 (52.2)
Non-R0 resection, n (%)	22 (47.8)
Major complication (≥3), n (%)	8 (17.4)
Recurrence, n (%)	23 (50.0)
Death, n (%)	23 (50.0)

**Table 3:** Comparison according to HALP group.

Variable	Low HALP (n = 23)	High HALP (n = 23)	*p*
Tumor size (mm), median (IQR)	140 (112–210)	90 (75–130)	0.011
Non-R0 resection, n (%)	16 (69.6)	6 (26.1)	0.012
Major complication ≥ 3, n (%)	8 (34.8)	0 (0)	0.004
Recurrence, n (%)	15 (65.2)	8 (34.8)	0.048
Death, n (%)	16 (69.6)	7 (30.4)	0.017

**Table 4:** Univariable and multivariable Cox regression analyses for overall survival (OS) and disease-free survival (DFS).

Variable	OAS Univariable HR (95% CI)	*p*	OAS Multivariable HR (95% CI)	*p*	DFS Univariable HR (95% CI)	*p*	DFS Multivariable HR (95% CI)	*p*
High vs. Low HALP	0.29 (0.12–0.70)	0.006	0.82 (0.23–2.87)	0.785	0.39 (0.16–0.96)	0.040	0.74 (0.28–1.95)	0.559
Grade	2.73 (1.50–4.98)	0.001	2.23 (1.18–4.21)	0.014	2.28 (1.27–4.10)	0.006	1.94 (1.05–3.59)	0.033
Non-R0 resection	4.04 (1.58–10.32)	0.003	2.76 (0.89–8.56)	0.083	7.58 (2.54–22.64)	<0.001	7.22 (2.19–23.77)	0.002

## Data Availability

The datasets supporting the conclusions of this article are included within the article and its tables. Additional data are available from the corresponding author upon reasonable request.
